# Digital PCR for the Authentication of KAMUT^®^ Brand Wheat in Grain and Flour Mixtures

**DOI:** 10.3390/foods15050910

**Published:** 2026-03-06

**Authors:** Caterina Morcia, Roberta Ghizzoni, Raffaella Bergami, Sonia Scaramagli, Chiara Delogu, Lorella Andreani, Valeria Terzi, Ilaria Carrara, Della Della

**Affiliations:** 1 Council for Agricultural Research and Economics (CREA), Research Centre for Genomics and Bioinformatics (GB), Via S. Protaso 302, 29017 Fiorenzuola d’Arda, PC, Italy; roberta.ghizzoni@crea.gov.it (R.G.); valeria.terzi@crea.gov.it (V.T.); ilaria.carrara18@gmail.com (I.C.); della.della@teagasc.ie (D.D.); 2 Coop Italia, Via del Lavoro, 6/8, 40033 Casalecchio di Reno, BO, Italy; raffaella.bergami@coopitalia.coop.it (R.B.); sonia.scaramagli@coopitalia.coop.it (S.S.); 3 Council for Agricultural Research and Economics (CREA), Research Centre for Plant Protection and Certification (CREA-DC), Via Emilia km 307, 26838 Tavazzano, LO, Italy; chiara.delogu@crea.gov.it (C.D.); lorella.andreani@crea.gov.it (L.A.)

**Keywords:** molecular traceability, digital PCR, wheat, variety, allelic discrimination

## Abstract

Food safety, quality, and traceability have become increasingly important in the agrifood industry in recent years, necessitating the use of reliable and rigorous analytical tools to ensure agrifood surveillance. This work focuses on the development and application of a new molecular approach to verify the authenticity of a specific variety of *Triticum turgidum* ssp. *turanicum*, commonly known as Khorasan wheat, which is commercially sold under the KAMUT^®^ trademark. A method based on duplex digital PCR was developed to identify and quantify *T. turanicum* variety QK-77 used in KAMUT^®^ brand products. The assay was validated on pure QK-77 variety alone and mixed with other varieties and on other cereal species. The developed PCR-based assay, tested using two digital PCR platforms (cdPCR and pdPCR), has high sensitivity and accuracy and can be applied to quantify the QK-77 variety in commercial grain lots and processed foods.

## 1. Introduction

Wheat is among the top three cereal crops, with around 770 million tonnes being harvested annually [1].

About 95% of the wheat cultivated worldwide is bread wheat (*Triticum aestivum*). Most of the remaining 5% is durum wheat (*T. turgidum* var *durum*) with small proportions represented by “primitive” or “ancient” wheats such as einkorn (*T. monococcum*), emmer (*T. dicoccum*), spelt (*T. spelta*) and two subspecies of *T. turgidum* that share the same genomic constitution as *T. durum*. These are *T. polonicum* (Polish wheat) and *T. turanicum* (Khorasan wheat, named after the Iranian region where it was first described). Evidence suggests that *T. polonicum* and *T. turanicum* are closely related and that *T. polonicum* may have originated through hybridization with *T. dicoccon* [2]. Recently, there has been increasing interest in ancient wheats, accompanied by a growing consumer demand for artisanal and traditional products made from these locally cultivated varieties. Consumers are often willing to pay a premium for such products, partly because these “ancient grains” are perceived to be healthier alternatives to modern wheat due to the high nutritional value, in particular, protein, essential minerals and polyphenol content [3,4,5]. Another important factor driving this renewed interest is the distinctive sensory profile of ancient wheats [6]. Many of these varieties are valued for their richer, more complex flavor compared to modern bread and durum wheats. As a result, several of these grains have been reintroduced into niche and specialty markets. However, the scientific evidence supporting the superior health benefits of ancient wheats remains inconclusive, with studies producing highly debatable and sometimes contradictory results [7,8,9,10,11].

While their nutritional advantages have yet to be clearly demonstrated, ancient wheats offer other valuable attributes. In particular, they can play a role in agricultural systems and rural economies—for example, by supporting biodiversity, promoting low-input and sustainable farming practices, and creating market opportunities for small-scale and local producers [12]. Polish and Khorasan wheats, that can be cultivated organically and are resilient to stressful environmental conditions, represent appealing and alternative crops for the Mediterranean basin’s marginal areas. Thanks to their adaptability to arid soils and harsh climatic conditions, they often thrive where modern varieties struggle; however, their tall stature makes them more susceptible to lodging. Nonetheless, they are a source of genetic diversity and have been grown by traditional (mainly local) farmers for use in low-input agriculture [13]. In addition, ancient wheats are significant economically because they cater to a particular customer niche while being mindful of the need to preserve cultural traditions that are frequently connected to a healthier way of life. In brief, while using ancient wheats has drawbacks—low yields (about 25–30% less than soft wheat and 15–20% less than durum wheat [12]), a propensity to lodge, and relatively poor dough workability—it also offers opportunities throughout the value chain to support biodiversity conservation and enhancement as well as to produce a range of specialty goods with unique sensory qualities.

The ancient KAMUT^®^ brand wheat (*Triticum turgidum* ssp. *turanicum*) has become increasingly popular in recent decades [14,15] thanks to its reputation for health benefits [16,17]. Following its success, other wheat varieties have also grown in importance and are now widely marketed, especially in Western countries. The KAMUT^®^ trademark, registered in 1990 for the variety “QK-77”, is mainly used for products such as pasta, noodles, granola, puffed cereals, and breads. Europe is now the key market, with Italy importing about 70% of all KAMUT^®^ wheat and playing a leading role in developing new cereal-based products. Today, KAMUT^®^ products are sold not only in supermarkets across Italy but also in health-food and specialty organic stores [18].

Because of the economic value gained by specific accessions of Khorasan there is interest in the development of analytical approaches capable of ascertaining and verifying the actual identity of genotypes from field to the final products. The greater commodity value of particular genotypes could in fact lead to substitutions with wheats of lower commercial value and therefore to fraud. Although there is no unified definition of “food fraud” in European law, food fraud is about “any suspected intentional action by businesses or individuals for the purpose of deceiving purchasers and gaining undue advantage therefrom, in violation of the rules referred to in Art 1(2) of Regulation (EU) 2017/625 (The Agri-Food Chain Legislation)”. Specifically, substitution is referred to as the action of replacing an ingredient, or part of a product, of high value with another ingredient or part of a product of lower value.

Among the analytical techniques that can be used, DNA is a perfect target for food-authenticity purposes for many reasons. First, DNA is a universal molecule; the majority of cells in organisms contain DNA, which may make it possible to extract the same information from any sample taken from the same source, regardless of the tissue of origin. Second, DNA is highly stable, so it is more resistant to physical and chemical industrial processes. For example, DNA is not affected by environmental factors (e.g., soil composition, climate condition, extraction methods, stage of ripeness, harvesting time, and storage period). On the other hand, the metabolic profile, including phenols, sterols, triacylglycerol, free fatty acids, trace elements, etc., can be severely impacted by those factors. Consequently, metabolite analysis (e.g., IR spectroscopy, NMR, various chromatographic methods, etc.) combined with chemometric methods are often insufficient to discriminate [19]. Third, due to the degeneration of the genetic code and the presence of many non-coding regions, DNA can supply more information than protein through the collection of sequence data [20]. Fourth, DNA is detectable in low concentrations, so it permits the detection of low concentrations of adulterants. As a result, DNA markers, particularly in PCR-based methods, have quickly surpassed all other tools in the field of food authenticity for the detection of food fraud [20,21].

In the wheat sector, there are already examples of analytical systems based on informative DNA sequences for the identification and quantification of both wheat species and modern varieties. The distinction between hexaploid and tetraploid wheats is reported by Morcia et al. [22] and applied at an industrial level for internal control. DNA-based assays are also capable in discriminating between naked and hulled wheats at different ploidy levels [23]. Finally, dual approaches based on digital PCR and LAMP for, respectively, quantification and point-of-care identification of modern durum wheat varieties have been developed by Morcia et al. [24] and Cibecchini et al. [25].

At a higher level of complexity there is the possibility of tracing accessions of obsolete wheats, characterized by high levels of genetic heterogeneity in comparison with modern varieties. The present study takes place within this sector, stimulated by the interest of industry, large-scale retail trade and consumers in the possibility of also verifying genetic authenticity for wheat landraces.

The objective of this work was the development and evaluation of a DNA-based approach based on digital PCR for traceability and identity preservation along the production chain, starting from raw materials.

The approach was applied to the traceability of the specific variety of *Triticum turgidum* ssp. *turanicum* QK-77. In detail, sets of SNP markers that characterize the variety have been identified with respect to the germplasm of tetraploid wheat. Starting from this information, highly specific digital PCR assays have been developed to enable precise quantification of the QK-77 variety directly in grain and flours. The study emphasizes how crucial the traceability system is to ensure food quality and authentication, especially in high-value markets such as monovarietal products. Intentional or inadvertent adulteration, along with the resulting fraud, is a worldwide source of economic fraud for consumers and for all parties engaged in food production and distribution [21].

## 2. Materials and Methods

### 2.1. Samples

Three groups of samples were used in this study.

In the first step, the analyses were done using certified seeds of a tetraploid wheat, hexaploid wheat and barley working collection obtained from the Council for Agricultural Research and Economics—Genomics and Bioinformatics (CREA-GB, Italy), from the Council for Agricultural Research and Economics—Cereal and Industrial Crops (CREA-CI, Italy), from the Council for Agricultural Research and Economics—Animal Production and Aquaculture (CREA-ZA, Italy) and from the Council for Agricultural Research and Economics—Centre of Defense and Certification (CREA-DC, Italy). QK-77 certified seeds, i.e., the variety sold under the KAMUT^®^ brand, were obtained from Fort Collins seed vault, Colorado State University, USA (Table 1). All certified seeds and accessions belonging to the species *Triticum turgidum* L. subsp. *turanicum*, *Triticum turgidum* L. subsp. *polonicum*, *Triticum timopheevii* and *Triticum turgidum* L. subsp. *durum* have previously been verified with SSR molecular markers to test their homogeneity, distinctiveness, and species.

To assess the robustness of the method, the analyses were applied to a second set of samples consisting of mixtures prepared with different proportions of QK-77 flour and another durum wheat variety distinct from QK-77 (Claudio variety). This set included pure 100% QK-77 flour, as well as blends containing 97% QK-77 and 3% Claudio, 95% QK-77 and 5% Claudio, 90% QK-77 and 10% Claudio, 75% QK-77 and 25% Claudio, and 50% QK-77 and 50% Claudio, together with a sample composed entirely of the Claudio variety. To prepare these mixtures, each flour was first weighed separately to obtain the exact amounts required to reach the desired percentages, and the components were then combined to achieve a final total weight of 100 g.

The third set included pasta and bread samples prepared with different percentages of QK-77 flour and non-KAMUT^®^ commercial semolina flours. Two pasta samples were prepared by mixing tap water and flours (90% QK-77 flour and 10% commercial non-KAMUT^®^ semolina flour). The samples were dried in an oven at 80 °C for 1 h, followed by 3 h at decreasing temperature. This desiccation thermal profile is commonly used for commercial pasta preparation. Two bread samples were prepared by mixing tap water and flours (93% QK-77 flour and 7% commercial non-KAMUT^®^ semolina flour). The samples were prepared in a bread machine (MD 11011, Medion) by mixing 240 g flour, 180 mL water, 5 g dehydrated yeast, and 5 g salt and following a standardized sequence of 4 h including kneading, rising, and baking at 130 °C.

### 2.2. DNA Extraction and Quantity/Quality Evaluation

The seeds were milled using a Cyclotec (FOSS Italia S.r.l., Padova, Italy) at a 0.2 mm grid diameter, avoiding any contamination between samples. Genomic DNA was extracted from 100 mg of milled samples and mixed flours using a DNeasy Plant Mini Kit (Qiagen, Milan, Italy) according to the manufacturer’s instructions. Pasta and bread samples were milled with an M20 Universal Mill (IKA) and DNA was extracted from 2 g of milled samples using a DNeasy Mericon Food Kit (Qiagen, Milan, Italy) according to the manufacturer’s instructions.

The evaluation of the quantity and quality of the extracted DNA was performed using a Qubit™ fluorometer in combination with the Qubit™ ds DNA-BR Assay kit (Invitrogen by Thermo Fisher Scientific, Monza, Italy). DNA integrity was verified using 1% agarose gel electrophoresis stained with ethidium bromide (EtBr) and viewed in Gel Doc XR (BioRad, Hercules, CA, USA).

### 2.3. SNP Selection for Digital PCR Analysis

The SNP dataset developed from Diversity Arrays Technology sequencing (DArTseq) of Khorasan wheat variety “QK-77” certified seeds and other durum wheat varieties performed in a previous work [24] was used to select QK-77-specific molecular markers. A useful SNP as an allelic variant unique to QK-77 was identified and was mapped on the durum wheat genome sequence (Sequence ID: LT934111.1, *Triticum turgidum* subsp. *durum* genome assembly, chromosome: 1A) using the Basic Local Alignment Search Tool (BLAST2.17.0). The QK-77-specific SNP is localized on chromosome 1A of durum wheat, the reference allele is T (wildtype), and the alternative is C (mutant). The C allele is present only in the variety Khorasan variety “QK-77”, while the other durum wheat varieties tested were homozygous T/T.

### 2.4. Primer and Probe Design

**Figure 1:**
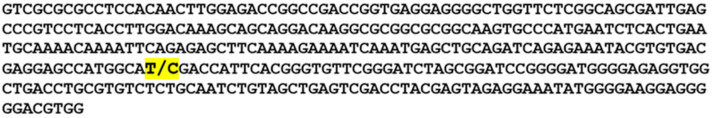
SNP sequences selected for the development of Assay ID ANH6UTV. The SNP molecular marker (highlighted in yellow) was identified by aligning the obtained sequence with the reference BLAST Sequence ID LT934111.1, *Triticum turgidum* subsp. *durum* genome assembly, chromosome: 1A.

Digital PCR assay, consisting of two primers and two MGB probes, was designed to amplify the selected private allele of Khorasan wheat variety “QK-77” certified seeds. The assay was designed based on the selected SNP sequence (Figure 1) using the Custom Assay design tool (Thermo Fisher Scientific, Monza, Italy) and is available as Assay ID ANH6UTV (Thermo Fisher Scientific, Monza, Italy). This custom assay is supplied at a 40× scale, with primer and probe concentrations of 36 µM and 8 µM respectively. The target allele for the “QK-77” variety was labeled with FAM, while the alternative allele was labeled with VIC.

### 2.5. Digital PCR Analysis

**Figure 2:**
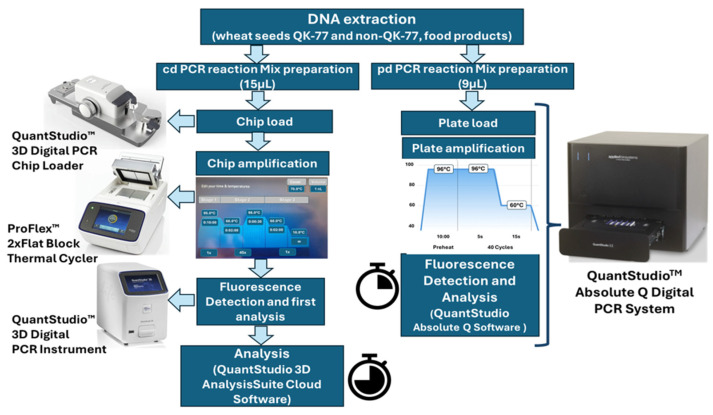
Workflow of cdPCR and pdPCR experiments.

For the analysis, two different digital instruments are used (Figure 2):

QuantStudio^TM^ 3D Digital PCR (Applied Biosystem by Life Technologies, Monza, Italia) system. It is a chip-based platform (cdPCR) consisting of three distinct units: a chip loader for automated sample partitioning and chip loading, a flat block thermal cycler for PCR amplification, and a fluorescence signal reader. Experiments were performed in duplicate. The reaction mixture (total volume of 16 μL) consisted of:

• 8 μL QuantStudio^TM^ 3D Digital PCR 2× Master Mix;
• 0.4 µL of Custom Assay ID ANH6UTV 40×, containing forward/reverse primers and VIC/FAM-MGB probes;
• 4 μL of DNA (10–20 ng/μL);
• nuclease-free water up to 16 μL.

A negative control containing nuclease-free water as a template was included in each analysis. A 15 µL volume of the reaction mixture was loaded onto the QuantStudio^TM^ 3D Digital PCR chips using the 3D Digital chip loader (Applied Biosystems by Thermo Fisher Scientific, Monza, Italy), following the manufacturer’s protocol. Amplification was performed in a ProFlex^TM^ 2Xflat PCR System Thermocycler (Applied Biosystems by Life Technologies, Monza, Italy) under the following conditions: 96 °C for 10 min, 45 cycles of 60 °C annealing for 2 min, and 98 °C denaturation for 30 s, followed by 60 °C for 2 min (final extension) and holding at 10 °C. Data were analyzed using the cloud-based platform QuantStudio^TM^ AnalysisSuite Cloud Software (v. 3.1.6). Each sample was analyzed in triplicate.

QuantStudio^TM^ Absolute Q Digital PCR (Applied Biosystem by Thermo Fisher Scientific, Monza, Italy) system. It is a plate-based digital PCR (pdPCR) platform and consists of a single instrument that consolidates all required steps into a 16-chip plate (MAP16 plate). Experiments were performed in duplicate. The reaction mixture (total volume of 9 μL) consisted of:

• 1.8 μL of Absolute Q™ DNA Digital PCR Master Mix 5×;
• 0.225 µL of Custom Assay ID ANH6UTV 40×, containing forward/reverse primers and VIC/FAM-MGB probes;
• 4 μL of DNA (10–20 ng/μL);
• nuclease-free water up to 9 μL.

A negative control containing nuclease-free water as a template was included in each analysis. A 9 µL volume of reaction mixture was loaded onto the MAP16 plate (Ref. A52688, Applied Biosystem by Thermo Fisher Scientific, Monza, Italy) and 15 µL of Isolation Buffer (Ref. A52730, Applied Biosystem by Thermo Fisher Scientific, Monza, Italy) was transferred to wells with the PCR mix. Amplification was performed under the following conditions: 96 °C for 10 min, 40 cycles of 60 °C annealing for 15 s, and 96 °C denaturation for 5 s. The files generated were analyzed using Absolute Q Software (v. 6.3). Each sample was analyzed in triplicate.

## 3. Results

### 3.1. DNA Extracted: Quantity and Quality Evaluation

**Figure 3:**
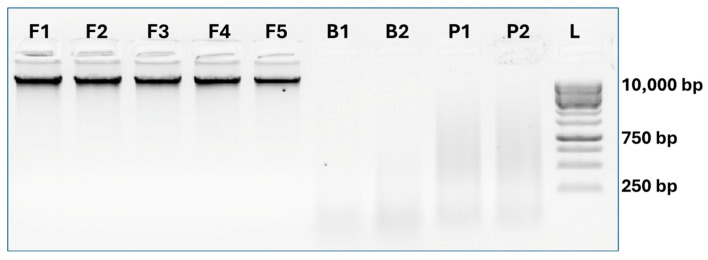
Agarose gel electrophoresis image of 100 ng of DNA extracted from flour (F1–F5), bread (B1, B2) and pasta (P1, P2). L = 4 µL GeneRuler 1 kb DNA Ladder (Thermo Scientific).

The concentration of genomic DNA extracted from seeds and mixed flours ranged from 103.7 to 153.4 ng/μL. In the case of bread and pasta, the obtained DNA was lower, likely due to food processing, with concentrations varying between 22.3 and 65.8 ng/μL. The DNA integrity was checked using 1% agarose gel electrophoresis. DNA extracted from flours (F1–F5) showed high molecular weight, whereas DNA obtained from processed food, bread (B1, B2) and pasta (P1, P2), exhibited a high fragmentation index (Figure 3).

### 3.2. Digital PCR Assay Specificity

**Figure 4:**
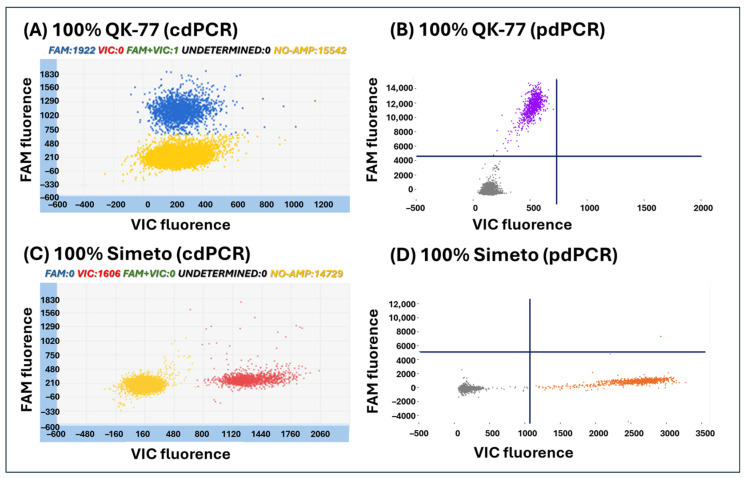
Two-dimensional scatter plots obtained by QuantStudio 3D-cdPCR (**A**,**C**) and Absolute Q-pdPCR (**B**,**D**) analyzing 40 ng of DNA extracted from 100% Khorasan wheat “QK-77” variety certified seeds (**A**,**B**) and from 100% Simeto variety (**C**,**D**). Blue and violet dots are QK-77 variety amplifications, red and orange dots are Simeto variety amplifications, yellow and gray dots are negative PCR partions without any target amplification.

The specificity of the developed dPCR assay was evaluated on QK-77 and on accessions/varieties listed in Table 1. As reported in the Materials and Methods, the mutant C allele located on chromosome 1A of the QK-77 variety was FAM-marked, while the wildtype T allele present in all other wheat varieties was VIC-marked. This assay effectively distinguishes the QK-77 variety from other durum wheat and tested species. Specifically, when the sample consists of 100% QK-77 variety, only the FAM signal is detected. Conversely, when the sample corresponds to any accession different from the QK-77 variety, the signal is VIC. Representative two-dimensional scatter plots obtained from both digital instruments are shown in Figure 4. Panels A and B display the results for 100% QK-77 DNA, while panels C and D show 100% Simeto variety DNA. In the 100% QK-77 sample, only the FAM signal was detected (blue for QuantStudio 3D-cdPCR and violet for Absolute Q-pdPCR). In the 100% Simeto sample (no QK-77 variety), only the VIC signal is detected (red for QuantStudio 3D-cdPCR and orange for Absolute Q-pdPCR). The yellow and black dots obtained in cdPCR and pdPCR, respectively, represent the PCR negative partitions without amplification.

### 3.3. Digital PCR Assay Sensitivity

When QK-77 flour is mixed with different amounts of other wheat, both FAM and VIC signals are detected. Table 2 reports the mean copies/µL of QK-77 and Claudio obtained by digital PCR assay in samples consisting of different mixtures of flours of the two genotypes (QK-77 and Claudio) and standard deviation (SD). The percentage of QK-77 present in each mixture was calculated using the following formula:

$$\mathrm{QK}\text{-}77\%=\frac{\mathrm{FAM}}{\mathrm{FAM}+\mathrm{VIC}}\cdot 100$$

**Figure 5:**
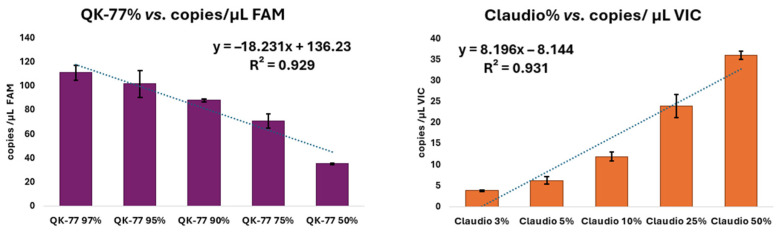
Regression curves obtained by plotting different concentrations of (**a**) QK-77 (violet histograms) and (**b**) Claudio (orange histograms) against copies/µL of FAM and VIC found, respectively. Error bars are reported.

Linearity was evaluated by plotting the expected concentrations of QK-77 and Claudio against the found copies/µL of FAM and VIC, respectively (Figure 5).

By exploiting the VIC channel as the contaminant-specific readout, the method provides a highly discriminative signal: no amplification is observed in the QK-77 reference, whereas a clear and reproducible positive response is detectable at contamination levels as low as 3% (*w*/*w*) of the Claudio variety. This demonstrates the assay’s strong capability to identify very low-level non-QK-77 contamination with high confidence. All flour samples were analyzed in triplicate using both digital platforms (cdPCR and pdPCR); the resulting measurements showed excellent agreement between instruments, with a correlation coefficient of R = 0.983.

### 3.4. Digital PCR Assay Applicability

**Figure 6:**
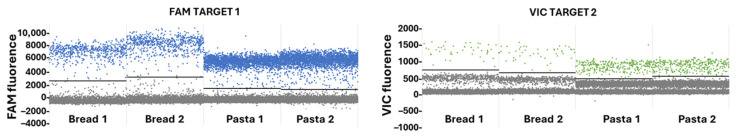
One-dimensional scatter plot obtained by Absolute Q-pdPCR analyzing 80 ng of bread and pasta DNA. Blue and green dots are positive PCR partitions for QK-77 and non-QK-77 respectively; gray dots are negative PCR partitions without any target amplification. Processed food samples were analyzed in triplicate.

To verify the applicability of the method, the developed dPCR assay was used to analyze processed samples, bread and pasta, prepared with known concentrations of QK-77 and commercial flour. Figure 6 shows the one-dimensional scatter plots obtained by Absolute Q-pdPCR. Table 3 reports the mean copies/µL of QK-77 (FAM) and non-QK-77 (VIC) detected in processed foods samples consisting of different mixtures of QK-77 and non-KAMUT commercial semolina flour. The correlation between expected and observed values showed a correlation coefficient of R = 0.986. This result indicates that the method performs effectively and reliably even when applied to processed food products.

## 4. Discussion

Food safety, quality, and traceability have become increasingly important in the agrifood industry in recent years, necessitating the use of reliable and precise analytical instruments to ensure effective surveillance. DNA-based technologies, with their superior sensitivity and throughput capacity, offer robust solutions for verifying product authenticity. A considerable amount of scientific literature and successful applications demonstrate that DNA-based techniques (e.g., real-time PCR (qPCR) or LAMP approaches, HRM, SSR or SNP molecular markers, DNA barcoding, genome sequencing, etc.) are often the most suitable choices in terms of rapidity, repeatability, efficiency, reproducibility, and accuracy [26]. The development of new molecular technologies able to verify food authenticity is essential for the food market, providing substantial benefits to consumers, farmers, the private sector, and governments [27].

KAMUT^®^ brand wheat represents a high commodity value product, and its market price is generally higher than those of other products derived from standard durum wheat or common wheat. Consequently, it is important to have a method applicable in the production chain to guarantee its authenticity and protect consumers from food fraud. dPCR, currently the gold standard for GMO quantification [28], finds, in the present work, an effective application for the traceability of a specific genotype.

The idea behind the creation of molecular tools for QK-77 variety identification is based on the potential to improve monovarietal flours and product control to reveal any fraud and confirm the authenticity of products in the supply chain.

Because of the rise in fraud incidents in the food industry, consumer demand for transparency is increased [29]. Greater transparency, in fact, equates to greater consumer confidence [30]. In previous works, a duplex cdPCR assay was successfully used in identification and quantification of common wheat along the pasta production chain. The limit of detection of this proposed method was 0.3% of common wheat contamination [22]. Ramos-Cabrer et al. [31] used droplet dPCR (ddPCR) for the traceability of the local wheat variety “Caaveiro” in flour mixtures.

To accurately identify products based on QK-77, a dPCR assay, based on a single-SNP discriminating method, was developed. This assay was designed as a duplex test to quantify the QK-77 variety and the other durum types simultaneously. Compared to conventional molecular techniques like qPCR, dPCR offers several advantages [32]. A primary benefit is the ability to quantify the target in an absolute manner without reference samples or calibration curves, whereas qPCR results remain relative to a standard curve [33]. Secondarily, even at very low target copy counts, accurate findings are ensured by strong sample partitioning (>20,000), which also permits the detection of rare targets in the presence of a significant background of non-target DNA [34]. Opinions about the relative commercial costs of dPCR and qPCR assays are contentious. Demeke and Dobnik [34] proposed that multiplexing can increase processivity; this is also related to the variety of instruments that are readily available on the market.

Actually, because modern dPCR machines allow for the reduction of reaction volumes and multiplexing targets (up to four in the case of QuantStudio Absolute Q), expenses can be lowered significantly. Furthermore, a significant simplification of the sample preparation and loading procedure has resulted in a considerably faster and simpler workflow (Figure 1). The cost estimate for the quantitative identification of a specific wheat genotype in a single sample is presented in Table 4.

The selected SNP as the basis for the developed test was validated both in the laboratory across all accessions/varieties listed in Table 1 and in silico using the available databases. Although the possibility of no specificity arising in non-tested accessions cannot be completely excluded, the panel used for SNP validation is sufficiently broad to cover what is currently and realistically present on the market. The choice to focus on a single SNP is justified by the need to minimize analytical steps and, consequently, reduce overall costs and processing time. Several examples from previous work support the effectiveness of this approach [35,36].

## 5. Conclusions

The dPCR assay, designed on a selected sequence and based on a single-SNP discriminant method, proved effective for simultaneously quantifying the QK-77 variety and other wheats. A key result is the strong discriminatory ability against non-QK-77 material, exploiting the VIC channel as a specific readout of the contaminant. The method showed no VIC signal in the QK-77 reference and demonstrated a clear and reproducible positive response at contamination levels as low as 3% (*w*/*w*) of non-QK-77 varieties. This demonstrates the method’s remarkable ability to detect very low levels of contamination, ensuring high reliability in the assessment of QK-77-based products.

The advantages of this new method, high precision, accuracy, sensitivity, and applicability, are fully confirmed. The results establish the dPCR assay as a valid tool to ensure the purity of wheat and may pave the way for further use of this technique in different production chains. The food industry and merchants have recognized the potential of this methodology and have worked together to develop and validate these technologies. Our analysis confirms that this technique offers significant savings in terms of reagents and analytical time. This is made even more evident by the most recent developments in dPCR instruments, which enable significant analytical cost savings by providing greater analytical flexibility and scalability. As far as we are aware, this is the first study focusing on using dPCR to measure the QK-77 variety in flours and processed foods prepared with KAMUT^®^ brand wheat. Thus, the application of the developed cdPCR and pdPCR assay to identify and quantify the QK-77 variety is a novelty. The assay’s specificity was confirmed through extensive in silico and laboratory testing across a representative variety panel (Table 1); the focus on a single SNP ensures a streamlined, cost-effective workflow. This assay is rapid and cost-effective, making it particularly suitable as a control tool at multiple stages of the supply chain, from wheat production to commercial products. It represents a practical support to the current control framework, which is still largely based on documentation rather than analytical evidence. Moreover, this approach lends itself to future technological developments. For instance, “point-of-care” formats based on naked-eye interpretation of analytical results, already demonstrated for a durum wheat variety [25], show that reliable genotyping can be performed without the need for dedicated laboratory equipment. The same concept could be extended to the present assay, providing portable tools for rapid on-site verification.

Overall, this study represents an important step forward for the agrifood traceability sector. By offering a reliable, sensitive, and scalable solution for verifying QK-77 variety authenticity, the developed dPCR approach supports industry, regulators, and consumers alike, contributing to the protection of high-value grain markets and promoting greater integrity in the global food system.

## Figures and Tables

**Table 1:** Certified seeds and accession used along the study.

Species	Number of Accessions	Accession Identifier	Provider
*Triticum turgidum* L. subsp. *turanicum* (Jakubz.) Á.Löve & D.Löve	1	QK-77	Fort Collins seed vault, Colorado State University, USA
*Triticum turgidum* L. subsp. *turanicum* (Jakubz.) Á.Löve & D.Löve	12	CLTR11390, PI167481, PI191599, PI192641 (PI192611), PI254206, PI278350, PI306665, PI576854, PI624429, PI192658, Kamut Mulino De Vita, Perciasacchi	Research Centre CREA-CI, Foggia, Italy
*Triticum turgidum* L. subsp. *turanicum* (Jakubz.) Á.Löve & D.Löve	3	FAR 279, FAR 280, FAR 295	Research Centre CREA-ZA, Lodi, Italy
*Triticum turgidum* L. subsp. *polonicum* (L.) Thell	3	FAR 281, FAR 296, FAR 297	Research Centre CREA-ZA, Lodi, Italy
*Triticum timopheevii* (Zhuk.) Zhuk	1	W899	Research Centre CREA-DC, Tavazzano (LO), Italy
*Triticum spelta* (L.)	7	Benedetto, Forenza, Giuseppe, Maddalena, Pietro, Rita, Rossella	Research Centre CREA-DC, Tavazzano (LO), Italy
*Triticum turgidum* L. subsp. *durum* (Desf.) Husn.	28	Achille, Antalis, Anvergur, Aureo, Babylone, Bronte, Claudio, Core, Iride, Fabulis, Kyle, Levante, Maestrale, Marco Aurelio, Miradoux, Monastir, Navigator, Normanno, Odisseo, Orizzonte, Pigreco, Relief, Rusticano, Saragolla, Senatore Cappelli, Simeto, Svevo, Tyrex	Research Centre CREA-GB, Fiorenzuola d’Arda (PC), Italy
*Triticum aestivum* (L.)	14	Apulia, Criterium, Extrem, Solehio, Alampur, Algeri, Anversa, Monviso, Rebelbe, Antelao, Azzurra, San Domino, Winner, Solina	Research Centre CREA-GB, Fiorenzuola d’Arda (PC), Italy
*Hordeum vulgare* (L.)	8	Morex, Andreea, Dana, Madalin, Fior868, Tiffany, Rodoz, Tremois	Research Centre CREA-GB, Fiorenzuola d’Arda (PC), Italy

**Table 2:** Digital PCR results obtained analyzing samples consisting of different percentages of QK-77 and Claudio flours. Mean copies/µL FAM (QK-77) and VIC (Claudio) are reported. The flour samples were analyzed in triplicate and standard deviations (SDs) are provided.

Flour Mix	Mean Copies/µL FAM ± SD	Mean Copies/µL VIC ± SD	Mean % QK-77 Found ± SD
100% QK-77	126.43 ± 7.02	0 ± 0	100 ± 0
97% QK-77 + 3% Claudio	111.12 ± 6.1	3.9 ± 0.2	96.6 ± 0.8
95% QK-77 + 5% Claudio	101.86 ± 11.31	6.3 ± 0.89	94.11 ± 1.39
90% QK-77 + 10% Claudio	88.28 ± 1.18	11.98 ± 1.12	88.07 ± 0.85
75% QK-77 + 25% Claudio	71.03 ± 5.91	24.02 ± 2.72	74.75 ± 0.57
50% QK-77 + 50% Claudio	35.38 ± 0.8	36.02 ± 0.91	49.56 ± 0.06
100% Claudio	0 ± 0	99.96 ± 8	0 ± 0

**Table 3:** Results obtained by digital PCR assay in bread and pasta samples consisting of different percentages of QK-77 genotype. Mean obtained FAM copies/µL of QK-77 and VIC of other varieties are reported. The samples were analyzed in triplicate and standard deviations (SDs) are reported.

Food Product	Mean Copies/µL FAM ± SD	Mean Copies/µL VIC ± SD	Mean % QK-77 Found ± SD
Bread 1 (93% QK-77)	74.78 ± 8.75	5.18 ± 0.09	93.48 ± 0.61
Bread 2 (93% QK-77)	64.62 ± 5.67	4.46 ± 0.98	93.58 ± 0.8
Pasta 1 (90% QK-77)	316.15 ± 42.44	36.04 ± 2.9	89.89 ± 0.27
Pasta 2 (90% QK-77)	252.18 ± 37.58	31.02 ± 4.12	88.92 ± 0.16

**Table 4:** Comparative analysis of operational parameters and costs for the quantitative detection of a specific wheat genotype using cdPCR and pdPCR platforms.

Parameters Considered	QuantStudio 3D (cdPCR)	QuantStudio Absolute Q (pdPCR)
Cost per reaction	About EUR 10	About EUR 5
Reaction volume	15–16 µL	9 µL
Complexity in workflow	More complex (manual upload)	Low complex (auto-upload)
PCR and analysis time	3–3.5 h	1.5 h
Possibility of multiplexing	2 targets	4 targets

## Data Availability

The original contributions presented in the study are included in the article, further inquiries can be directed to the corresponding author.
